# Complete Genome Sequence of Campylobacter fetus subsp. *testudinum* Strain Pet-3, Isolated from a Lizard (Hydrosaurus pustulatus)

**DOI:** 10.1128/genomeA.01420-14

**Published:** 2015-02-19

**Authors:** Chao-Min Wang, Zong-Yen Wu, Wei-Yau Shia, Yi-Jyun Jhou, Kwong-Chung Tung, Ching-Lin Shyu

**Affiliations:** a Research Center for Biodiversity, China Medical University, Taichung, Taiwan; b Department of Veterinary Medicine, College of Veterinary Medicine, National Chung-Hsing University, Taichung, Taiwan; c Graduate Institute of Microbiology and Public Health, College of Veterinary Medicine, National Chung-Hsing University, Taichung, Taiwan

## Abstract

The whole-genome sequence for Campylobacter fetus subsp. *testudinum*, a pathogen isolated from humans and turtles, has been reported recently. We present another completed genome sequence of the C. fetus subsp. *testudinum* strain pet-3, which was isolated from a lizard in Taiwan, for further genomic comparison study.

## GENOME ANNOUNCEMENT

Campylobacter fetus is an important human pathogen with a diverse host range, including mammals, fowls, and reptiles (1–3). Previous studies demonstrated that strains of C. fetus isolated from reptiles were genetically distinct from mammal-associated C. fetus by multilocus sequencing typing (4, 5). The genetic distance between mammal- and reptile-associated C. fetus is larger than within mammal-associated C. fetus. Recently, a polyphasic study was undertaken to determine the taxonomic position of those strains isolated from reptiles and humans (6). The results, including a whole-genome sequence, showed that those strains are closely related to C. fetus but are clearly different from recognized subspecies of C. fetus. Therefore, a novel subspecies, C. fetus subsp. *testudinum*, is proposed (7). Here, we report a whole-genome sequence of C. fetus subsp. *testudinum* strain Pet-3, which was isolated from a lizard (Hydrosaurus pustulatus), for further genomic comparison study.

Whole-genome paired-end sequencing was performed on an Illumina MiSeq desktop sequencer (Illumina Co., USA). A total of 14,590,400 reads were generated, resulting in 2,045-fold sequencing coverage. After end-trimming by Q20 cutoff, the remaining reads were *de novo* assembled into 35 contigs using CLC Genomics Workbench version 6.5.1. By comparing the genome sequences from related species using MUMmer version 3.23 and further bioinformatics analysis using GapCloser version 1.12-r6, these 35 contigs were manually inspected and concatenated into a single scaffold of a draft genome sequence using Consed version 26. The remaining six gaps within the scaffold were further closed by PCR walking and Sanger sequencing.

The whole-genome size of C. fetus subsp. *testudinum* Pet-3 is 1,776,391 bp, with an average G+C content of 33.13%. The open reading frames (ORFs) were predicted using Glimmer version 3.02 and prokaryotic GeneMark.hmm version 2.10f. The rRNA (rRNA) and tRNA (tRNA) genes were identified by RNAmmer version 1.2 and tRNAscan-SE version 1.23 software, respectively. The genome contains 1,796 putative protein-coding genes, 3 rRNA operons, and 43 tRNA genes. The putative functions of the genes were annotated against the NCBI nr (nonredundant), microbial RefSeq protein, and COG (Clusters of Orthologous Groups for Unicellular clusters) databases, as well as the KEGG (Kyoto Encyclopedia of Genes and Genomes) protein database using BLASTp. BLAST analysis indicated a high degree of similarity between the reptile-associated C. fetus subsp. *testudinum* genomes. A clustered regularly interspaced short palindromic repeats (CRISPR)–Cas system, an S-layer coding region, and a putative tricarballylate catabolism pathway were presented as predicted by protein analyses. Additionally, based on the core proteomes, 99% to 100% amino acid identity was observed between the proteomes common to C. fetus subsp. *testudinum* strain 03-427 and strain Pet-3. The most variable region between two strains was observed in the coding region of surface array protein A, with only 83% to 91% similarity. Further study on comparing the whole-genome sequence between C. fetus subsp. *testudinum* strains can provide a better understanding of the host virulence, adaptation evolution, and the taxonomic structure for those reptile-associated Campylobacter subspecies.

### Nucleotide sequence accession number.

The complete genome sequence of C. fetus subsp. *testudinum* strain Pet-3 has been deposited in GenBank under the accession number CP009226.
